# Supportive Care for Superutilizers of a Managed Care Organization

**DOI:** 10.1089/jpm.2019.0288

**Published:** 2020-10-14

**Authors:** Ritabelle Fernandes, Ed G. Fess, Sandy Sullivan, Mona Brack, Tara DeMarco, Dongmei Li

**Affiliations:** ^1^ Division of Palliative Medicine, Department of Geriatric Medicine, John A Burns School of Medicine, University of Hawaii, Honolulu, Hawaii, USA.; ^2^ Bristol Hospice Hawaii, LLC, Honolulu, Hawaii, USA.; ^3^ Ohana Health Plan, Inc., Honolulu, Hawaii, USA.; ^4^ University of Rochester School of Medicine and Dentistry, Rochester, New York, USA.

**Keywords:** chronic advanced illness, hospice, pacific islander, palliative care, superutilizers, supportive care

## Abstract

***Background:*** Ohana Health Plan, Inc., (OHP) is one of the first managed care organizations offering supportive care services targeted to superutilizers. Bristol Hospice Hawaii, LLC, partnered with OHP to provide interdisciplinary supportive care services to home-bound OHP members.

***Objectives:*** The purpose of this study was to measure symptom relief, satisfaction, resource utilization, and cost savings associated with supportive care.

***Design:*** Prospective study.

***Setting:*** Over 12 months, 27 superutilizer members residing in the community were referred by OHP, 21 members were enrolled into supportive care.

***Measurements:*** Data were collected upon admission and repeatedly thereafter using the Edmonton Symptom Assessment Scale (ESAS) and the Missoula–Vitas Quality of Life Index (MVQOLI). The Family Satisfaction with Advanced Cancer Care (FAMCARE) Scale was administered at discharge. Emergency department (ED) visits and hospital utilization were tracked.

***Results:*** Median age was 63 years; more than half had cardiac diagnoses. Majority of members were Hawaiian and other Pacific Islander. Median length of stay in supportive care was 90 days. Five (23%) members enrolled in hospice following supportive care. Symptom improvement occurred in pain (*p* < 0.0001), anxiety (*p* = 0.0052), and shortness of breath (*p* = 0.0447). This model has shown a 79.5% reduction of ED visits per thousand members and a 75% reduction of hospitalizations per thousand. Overall net savings was 36%. Discussions and documentation of end-of-life wishes increased from 23% to 85%.

***Conclusion:*** Supportive care is highly effective in reducing costs associated with superutilizers. Our experience demonstrates the effectiveness of supportive care approaches in this population through improved care and lower health care costs overall.

## Introduction

In the United States, it is estimated that the top 1%, ranked by their health care expenses, accounted for 22.7% of total health care expenditures with an annual mean expenditure of $97,956.^1^ National health spending is projected to grow at an average of 5.5% per year for 2017–2026 to reach $5.7 trillion by 2026.^2^ The overall health share of U.S. gross domestic product is expected to rise from 17.9% in 2016 to 19.7% in 2026.^2^

There is growing evidence of the effectiveness and cost savings associated with palliative care.^3–6^ According to the National Consensus Project, palliative care may be defined as patient- and family-centered care that optimizes quality of life by anticipating, preventing, and treating suffering. Palliative care throughout the continuum of illness involves addressing physical, intellectual, emotional, social, and spiritual needs and facilitating patient autonomy, access to information, and choice.^7^ The American College of Clinical Oncology recommends that patients with advanced cancer should receive dedicated palliative care services early in the disease course and concurrent with active treatment.^8^

However, palliative care has traditionally been delivered late in the course of the disease to patients who are hospitalized in specialized inpatient (IP) units or as a consultative service for patients with uncontrolled symptoms.^9^ In the United States, there is a lack of national policy and payment mechanisms for palliative care, which has contributed to preventable suffering and low-value care.^10^ This is especially true for palliative care services outside of IP acute care settings.

Supportive care may be known as palliative care; the two terms are often used interchangeably. Supportive care is a commonly used term in oncology; however, no consensus definition exists.^11^ According to the National Cancer Institute, supportive care is care given to improve the quality of life of patients who have a serious or life-threatening disease.^12^ The goal of supportive care applied as early as possible in the course of disease is to prevent or treat the symptoms of a disease, as opposed to the disease itself. Supportive care is also aimed at lessening the side effects caused by treatment of a disease, as well as psychological, social, and spiritual problems related to a disease or treatment. Supportive care services have recently experienced remarkable growth and acceptance in oncology care, focusing on the patient experience from diagnosis through survivorship.^13^

Ohana Health Plan, Inc., (OHP) provides managed care services in Hawaii targeted toward government-sponsored health care programs, including Medicaid, Medicare, and Medicare Prescription Drug Plans.^14^ OHP is offered by WellCare Health Insurance of Arizona, Inc., (WellCare) and its mission is to improve lives of their members, including a commitment to quality care.

OHP designed a supportive care pilot program with the aim to improve quality of life and reduce suffering of its highest utilizing members with advanced chronic illness. Members and families were not included in designing the pilot program. This unique collaboration between a managed care organization and local hospice to offer supportive care services as a solution for high utilizing members with advanced chronic illnesses was based on related palliative care models in Hawaii between managed care organizations such as Blue Cross Blue Shield, University Health Alliance (UHA), Veterans Health Administration, and local hospices.^15–17^ The purpose of this study was to measure symptom relief, satisfaction, resource utilization, and cost savings associated with supportive care.

## Methods

A prospective design was used. Data were collected from participants and caregivers upon admission to the supportive care pilot program and then every week for 90 days. There was no control group; however, data on medical care utilization before service matriculation were collected retrospectively from each participant's utilization claims, and these findings provided a historical control.^18^ Participants (or their designated proxy) provided consent to participate in the supportive care pilot program with Bristol Hospice Hawaii, LLC, (BHH).^19^ Institutional Review Board approval was not sought or obtained as BHH has been providing supportive care services to members of other health plans in Hawaii and this was considered usual care.

### Sample

**FIG. 1.**
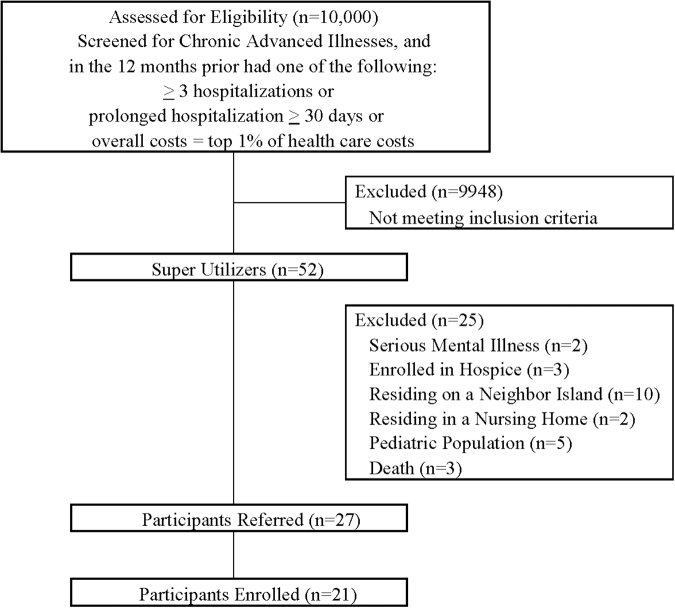
Referral flow diagram.

To identify superutilizers, OHP service coordinators (field-based care managers) referred members to the pilot program. OHP contracted BHH to provide supportive care to its identified members. Over a 12-month period (August 2017–August 2018), 27 members with advanced chronic illnesses were referred by their service coordinators to BHH (Fig. 1). The service coordination department had to preapprove members for the pilot program. These members were identified through claims utilization data as having greater than three hospitalizations in the past year or at least one prolonged length of stay (>30 days) or overall costs equivalent to the top 1% of health care costs in the population during the prior calendar year. Members with serious mental illness were excluded as OHP had other innovative case management programs in existence for this group. Members who resided in a nursing home or were terminally ill and enrolled in hospice were also excluded. BHH served adults on the island of Oahu; the pediatric population and those residing on other islands were excluded.

Diagnoses included varied advanced chronic illnesses and ranged from cardiovascular, pulmonary, renal, endocrine, and gastrointestinal diseases to oncological diseases. Twenty-one members (78%) agreed to be enrolled in the pilot program. Duration of supportive care services for the pilot program was preapproved for 90 days. Upon reaching the end of supportive care services at 90 days, members returned to their usual care or transitioned to hospice. The agreed-upon rate of reimbursement for the supportive care pilot program was the routine home hospice rate already in place.

### Intervention

BHH offered supportive care services using an interdisciplinary team approach. Hospice-type services and life-prolonging therapies were available concurrently, without members electing hospice benefit. The team comprised a hospice and palliative care board-certified physician, nurse practitioner, registered nurse, chaplain, social worker, certified nursing assistant, volunteer coordinator, and bereavement coordinator. The team was available 24 × 7 through phone and in-person to triage and provide symptom management.

Physicians and nurse practitioners performed a home visit on newly admitted participants and within one week of discharge from the hospital or an emergency department (ED) visit. The focus of the visit was symptom management and medication reconciliation. Registered nurses facilitated making provider appointments and coaching participants with demographic factors that are known to be associated with low health literacy.^20^ Many participants were immigrants and minorities whose native language was not English. Nurses performed two home visits weekly as well as accompanied participants to their appointments with the primary care provider and/or specialists, unless declined by the member or family. The care plan was communicated in person with the providers.

Skillful communication by social workers assisted with completion of physician orders for life-sustaining treatment (POLST) and advance health care directives (AHD). POLST was included in this pilot program as it was part of existing practice of supportive care. Advance care planning videos that are evidence-based video decision aids were also shown to participants.^21^ Copies of executed POLSTs and AHD were shared with all treating providers. The interdisciplinary care plan was individualized and member centric based on the member's goals of care. Medications related to the supportive care diagnosis were covered by BHH. Upon discharge from supportive care, the nurse provided OHP service coordinators with a detailed report, including all community resources and referrals provided to the member.

### Measures

Demographic data were collected by in-person self-report upon admission, including age, gender, ethnicity, diagnostic category, insurance status, and living arrangements. The Palliative Performance Scale (PPS) was used to measure performance status in palliative care.^22^

The Edmonton Symptom Assessment Scale (ESAS) was used to provide a clinical profile of symptom severity over time.^23^ ESAS assesses nine symptoms or conditions, including pain, tiredness, nausea, depression, anxiety, drowsiness, appetite, well-being, and shortness of breath. Each item is rated on a scale from 0 to 10, with 0 being the best option for each symptom or condition, for example, no pain, no fatigue, excellent appetite, and best well-being.

Missoula–Vitas Quality of Life Index (MVQOLI) was developed specifically to assess the multidimensional quality of life of patients who are aware of their terminal condition and understand that the goal of treatment is palliative.^24^ Five domains are assessed: symptoms, functioning, interpersonal relationships, well-being, and feelings of transcendence.

The Family Satisfaction with Advanced Cancer Care (FAMCARE) Scale was administered to caregivers upon discharge from supportive care services. It is a 20-item scale that assesses family satisfaction with care provided to patients who have an advanced cancer.^25^ ED visits, IP admissions, outpatient (OP) visits, and pharmacy costs were obtained through claims data during the study period.

### Analysis

Data were analyzed using statistical analysis software SAS, v9.4 (SAS Institute, Inc., Cary, NC). Summary statistics and frequency distributions were used to describe the characteristics of participants. Generalized estimating equation (GEE) models with the identity link function were used to assess the pre–post differences in clinical outcomes. Autoregressive 1 (AR1) variance–covariance structure was used to account for within-subject correlations. Linear contrasts were used to examine the mean longitudinal changes in clinical outcomes for all participants. Estimated coefficients and corresponding 95% confidence intervals from linear contrasts were used to quantify the differences in the GEE models.

To analyze change in utilization of ED visits and IP stays due to intervention, a per thousand members annualized methodology was adopted.^26^ This calculation enables direct comparison between different sized groups over varying time frames. The per thousand calculation is as follows: number of actual visits/member months*12000. Member months reflects the number of affected members each month, summed over the measured time frame. Direct costs of ED and IP visits were considered proprietary by OHP, but we obtained comparison per member per month (PMPM) cost data of this population before and after supportive care. Claims data were reviewed for three months before and after supportive care services were provided. Claims runout was rerun four months after the end of the pilot program to ensure a high rate of inclusion.

## Results

The demographic and clinical characteristics of the study population are summarized in Table 1. Of the 21 participants, 10 were males and 11 females. All had Medicaid, and nearly half were dual eligible, having Medicare coverage as well. Ages ranged from 31 to 92 years with the median age at 63 years. More than half had cardiac diseases, including congestive heart failure and uncontrolled hypertension. The second most common category was infectious diseases. Majority of participants were categorized as native Hawaiian, followed by Samoan and other Pacific Islander. Mean PPS was 50% at baseline and did not change over the 90 days of supportive care services. At the end of the study, 16 (77%) participants were still alive and 5 (23%) died. Five participants had transitioned to hospice. The mean length of stay in the supportive care program was 90 days based on the length of the pilot program.

**Table 1.** Demographics and Clinical Characteristics (*N* = 21)

Variable	Frequencies
Age, years	
<55	6 (28.6%)
55–64	6 (28.6%)
65–74	2 (9.5%)
75–84	5 (23.8%)
85+	2 (9.5%)
Gender	
Male	10 (47.6%)
Female	11 (52.4%)
Race/Ethnicity	
Hawaiian	8 (38.1%)
Samoan	4 (19.0%)
Other Pacific Islander	4 (19.0%)
Asian	4 (19.0%)
Portuguese	1 (4.9%)
Medicaid	
Yes	21 (100.0%)
No	0 (0.0%)
Medicare	
Yes	10 (47.6%)
No	11 (52.4%)
Diagnosis	
Cardiac	7 (33.3%)
Infectious	5 (23.8%)
Pulmonary	4 (19.1%)
Oncology	2 (9.5%)
Renal	1 (4.8%)
Endocrine	1 (4.8%)
Gastrointestinal	1 (4.8%)
Mean length of stay (days)	90
Palliative Performance Scale	
80%–100% (full ambulation & self-care)	2 (9.5%)
60%–70% (unable to work, poor ambulation)	6 (28.6%)
40%–50% (unable to work, mainly sits/lies)	10 (47.6%)
20%–30% (bed-bound, needs total care)	3 (14.3%)

The most common symptoms experienced by participants were pain and shortness of breath. ESAS score changes from baseline and end of study are shown in Table 2. Appetite, drowsiness, and tiredness worsened over time. There was statistically significant symptom control for pain (*p* < 0.0001), anxiety (*p* = 0.0052), and shortness of breath (*p* = 0.0447). Findings of the MVQOLI survey are shown in Table 3 for the 10 participants who completed the survey. The mean global quality of life was 3.90 of 5. Only 1 participant had an advance directive at baseline, this increased to 4 participants by the end of the pilot study. While 5 participants had completed the POLST at baseline, this increased to 18 participants by the end of the pilot study. Discussions and documentation of end-of-life wishes increased from 23% to 85% during the time of the study.

**Table 2.** Edmonton Symptom Assessment Scale Score at Baseline and End of Study

Variable	Baseline (*n* = 21)		Difference at end of study (*n* = 21)		
	Mean	SD	Mean	95% CI	p^*^
Pain	3.2857	0.8252	−3.21	−4.81 to −1.61	<0.0001
Tiredness	0.7619	0.3079	2.29	1.04 to 3.54	0.0003
Nausea	0.0476	0.0465	0.45	−0.25 to 1.15	0.2046
Depression	0.9048	0.4990	−0.90	−1.88 to 0.07	0.0698
Anxiety	1.0952	0.3921	−1.10	−1.86 to −0.33	0.0052
Drowsiness	0.6190	0.2472	2.68	1.85 to 3.50	<0.0001
Appetite	1.7143	0.4769	1.69	0.48 to 2.90	0.0061
Well-being	2.0476	0.5733	−0.66	−1.71 to 0.39	0.2168
Shortness of breath	1.3333	0.5055	−1.02	−2.02 to −0.02	0.0447

^*^ Analysis adjusted for within-subject correlations due to repeated measurements.

CI, confidence interval; SD, standard deviation.

**Table 3.** Quality of Life—Range of Responses and Means (*N* = 10)

Dimension	Possible range	Respondent range	Mean	SD	Median
Symptom	−5 to 11	1 to 10	5.60	3.03	6.00
Function	−5 to 11	0 to 7	3.90	2.33	4.50
Interpersonal	−5 to 11	1 to 11	5.90	3.81	5.50
Well-being	−5 to 11	0 to 10	6.30	2.95	7.00
Transcendent	−5 to 11	0 to 11	6.90	4.01	8.00
Total score	−25 to 55	12 to 44	28.60	8.88	30.50
Global score	1 to 5	2 to 5	3.90	0.74	4.00

**FIG. 2.**
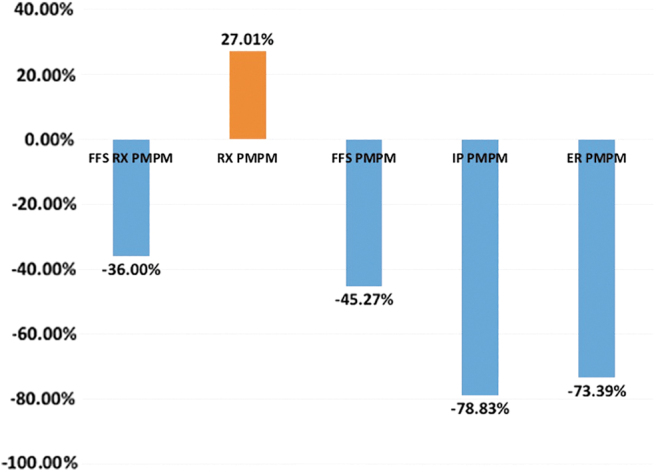
Percentage changes after versus before supportive care in hospitalization and emergency department visits costs PMPM (*n* = 21). FFS RX PMPM, overall net cost change (includes all patient care costs, including pharmacy). RX PMPM, pharmacy costs exclusively. FFS PMPM, fee for service (includes all patient care costs except pharmacy). IP PMPM, all costs associated with inpatient hospitalization. ER PMPM, all costs associated with emergency department visits. PMPM, per member per month.

Of the 21 members starting the program, there were 121 ED visits during the time up to a year before matriculation and 24 for the same time period after program completion. For these same members during the same time period, there were 65 IP admissions before the program and 13 afterward. Utilizing the per 1000 members annualized (/K) methodology described above, a direct comparison can be made. ED visits before supportive care were 5785/K, dropping to 1188/K afterward, a 79% decrease. The IP admission rate per 1000 members before supportive care was 3108/K, dropping to 772/K after supportive care, representing a 75% decrease. Looking at PMPM cost differences, supportive care in this population led to a reduction of ED visit costs by 73% and IP admission costs by 79%. During this period, there was a 113% increase in OP visit costs PMPM as well as a 27% increase in pharmacy costs on a PMPM basis. Due to the size of ED and IP cost reductions, the program realized a net cost reduction of 36% of the overall PMPM costs of these patients (Fig. 2).

Sixteen family caregivers completed the satisfaction survey upon discharge, as shown in Table 4. Satisfaction levels were generally high and stable, hence mean satisfaction scores were calculated for each caregiver. Mean item satisfaction ranged from 4.20 (the way tests and treatments are followed up by the treating physician) to 4.93 (the availability of nurses to the family). Lower satisfaction scores were associated with the treating physician's availability to the patient and their family, the way test and treatments were followed up by the physician, and the physician's attention to the patient's description of symptoms. Caregivers wrote comments such as *“I really appreciate all the program did for my father,” “Our nurse was so good and helped us with medicine and supplies,” “We were very unhappy with our primary care doctor and the lack of support. Ninety days go by too fast, and we don't want you to go. We really miss you guys,” and “We think this program is really helpful.”*

**Table 4.** Family Satisfaction with Advanced Cancer Care Satisfaction Survey (*N* = 16)

Variables	Mean	SD
The patient's pain relief	4.63	0.50
Information provided about the patient's prognosis	4.50	0.89
Answers from health professionals	4.63	0.81
Information given about side effects	4.88	0.34
Referral to specialists	4.62	0.65
Availability of a hospital bed	4.33	1.21
Family conferences held to discuss the patient's illness	4.62	0.87
Speed with which symptoms are treated	4.69	0.48
Doctor's attention to patient's description of symptoms	4.25	1.00
The way tests and treatments are performed	4.57	0.51
Availability of doctors to the family	4.25	1.18
Availability of nurses to the family	4.93	0.26
Coordination of care	4.88	0.34
Time required to make a diagnosis	4.56	0.53
The way family is included in treatment and care decisions	4.71	0.61
Information given about how to manage patient's pain	4.81	0.40
Information given about the patient's tests	4.62	0.51
How thoroughly the doctor assesses the patient's symptoms	4.31	1.01
The way tests and treatments are followed up by the doctor	4.20	1.01
Availability of the doctor to the patient	4.25	1.00

## Discussion

Superutilizers are characterized by lack of regular access to primary care, frequent ED visits and hospital IP admissions, multiple chronic health conditions, a history of mental health or substance abuse, low income and poverty, and low health literacy.^27–30^ Different strategies have attempted to reduce hospitalizations and readmissions by the use of case management, community health workers, navigators, and telemanagement programs.^31–36^ These programs have been successful, especially in dealing with chronic heart failure.

The Institute for Healthcare Improvement recommends the following for a smooth transition home from the hospital: enhanced admission assessment, effective teaching and enhanced learning, real-time patient- and family-centered handoff communication, and a posthospital care follow-up plan.^37^ Coordinated care and a clear discharge plan are needed to avoid rehospitalization, especially for superutilizers.^38^

The major challenge for providing effective supportive care is in the identification of members for whom targeted programs are most effective, thereby achieving desired outcomes. Managed care organizations in Hawaii have explored palliative care programs to reduce unnecessary ED visits, hospitalizations, and readmissions. In 2011, UHA Health Insurance, based in Honolulu, Hawaii, introduced a Concurrent Care model for members who have serious and chronic illnesses or life-limiting medical conditions.^16^ Qualifying conditions for their program include, but are not limited to, cancer, congestive heart failure, chronic obstructive pulmonary disease, kidney failure, Parkinson's disease, Alzheimer's disease, and amyotrophic lateral sclerosis. UHA contracts with local hospices to provide interdisciplinary palliative care.

The Hawaii Medical Service Association (HMSA), an independent licensee of the Blue Cross and Blue Shield Association, introduced a limited supportive care pilot program in 2013, which became a member benefit in 2017.^15^ This program is limited to persons with advanced cancer, congestive heart failure, and chronic obstructive pulmonary disease. Other conditions require prior approval from HMSA's medical director. Coverage is limited to 90 calendar days of service in a 12-month period. HMSA contracts with local hospices to provide supportive care. These programs at UHA and HMSA were not particularly targeted to superutilizers. Out pilot program is similar to UHA, in that all chronic advanced illnesses were included, and similar to HMSA, in that the pilot program was limited to a 90-day period.

BHH's interdisciplinary team provided symptom management and triage, reducing the need for IP hospitalization and ED visits. The unique intervention of nurses accompanying participants to primary care physician and specialist appointments was reported to be of value based on anecdotal feedback from the treating physicians. Family caregivers and participants appreciated the presence of a registered nurse who could provide symptom updates, share the care plan, and coordinate care, as evidenced by the FAMCARE Scale.

On reviewing health care costs overall, this small population saw a decrease in the traditional high-cost areas of IP and ED visits. Supportive care activities in this patient population were associated with a reduction of ED visit costs by 73% and IP admission costs by 79%, with a net savings of 36%. The costs for pharmacy saw an increase of 27% and may be explained, in large part, by improved medication compliance and attention to obtaining initial scripts. OP costs also rose (113%) and may be due to better compliance with OP visits of many types. While studies of larger populations are clearly needed to confirm these findings, it is encouraging to see that these interventions resulted in sufficient cost decreases of potentially unnecessary high-cost care to actually pay for the interventions.

Our findings were similar to those of Brumley et al., who found that within the Kaiser Permanente system, in-home palliative care significantly increased caregiver satisfaction while reducing costs of medical care at the end of life.^3^ Caregivers expressed greater satisfaction with nurses than with doctors (primary care physicians and specialists). High patient and caregiver satisfaction has also been found in similar populations with home-based palliative care services in Hawaii.^39^

Social workers provided many important services during the pilot study. They showed advance care planning videos to members and caregivers. They assisted with completion of the POLST, leading to discussions and documentation of end-of-life wishes, which reached a completion rate of 85% during the pilot study. The social workers focused on goals of care and what was truly important to the member. They also assisted with family reunification and travel, as well as long-term care planning and placement. One such example was a young participant with short gut syndrome and end-stage renal disease on hemodialysis. She was assisted in reuniting with her sisters in Salt Lake City, Utah, for Christmas. The social worker applied to the Dream Foundation for a one-way airline ticket, applied for Utah Medicaid, and collaborated with hospice and dialysis agencies in Utah to make this challenging interstate transfer a success.

The chaplains administered the MVQOLI; however, participants were immigrants and minorities whose native language was not English. They found the self-rating of their quality of life challenging, leading to a low completion rate of this survey. Chaplains provided a listening ear; many of these participants expressed anecdotally that they were never heard or listened to in the past by their health care team. The chaplain visits were especially of value to participants dealing with substance misuse. For some participants, seeking help from a mental health clinician was associated with stigma and few were estranged from their families. Having a trusted chaplain to talk with in the privacy of their homes helped them attain some measure of peace, self-worth, and meaning to their suffering.

While it quickly became evident that our work was received in a positive manner by the patients and their families, we were also interested to see if the outcomes were financially beneficial. Health plan adoption of this type of program will only occur if there is a net savings of all costs associated with improvement of these patient's lives. Instead of measuring only the savings from decreased ED and IP admissions, we wanted to reflect all costs, including those of BHH as the provider of services. Supportive care may be of growing interest as the United States transitions from fee-for-service to value-based care, payment transformation, and shared accountability systems.

Limitations and challenges encountered during the pilot study included billing issues, especially for dual eligible members, those who were eligible and receiving payment under both Medicare and Medicaid plans. BHH submitted claims in the pilot program using revenue code 651. OHP developed an internal indicator to identify members receiving supportive care services. OHP staff training had to be conducted for smooth processing of the supportive care claims. The sample size in this pilot study was small, only 21 members were enrolled. OHP agreed to approve 90 days of supportive care services, which BHH was aware of during the pilot study. Based on improvements in the many facets touched on by this study, OHP is considering an extension of supportive care services by 90-day intervals for members who need extra support.

This pilot study was conducted in a managed care setting, and results may not be generalizable. Another limitation was that the measurement tools used were not validated in a diverse population and ethnicities such as participants in the pilot program.

## Conclusions

In our study, supportive care was found to be an effective and welcomed service provided to superutilizers of a health plan by a contracted hospice provider in Hawaii. To the best of our knowledge, this study adds to the growing evidence base that palliative care improves quality of care and lowers costs. Advance care planning as evidenced by POLST completion and documentation of preferences had a significant improvement. There was improvement in symptom control of pain, anxiety, and shortness of breath, leading to reduced suffering of participants.

This model demonstrated success in reducing hospitalization and ED visits and in reducing the overall cost of care for the individuals identified as potentially benefiting from this service. Future studies examining how to best offer predictive modeling of patient selection are needed to identify superutilizers ahead of time. This would serve to bring supportive care services upstream before health care costs expand, allowing earlier care and improvement of these patients' lives.
